# Sleep quality is influenced by multiple factors and cannot be reduced to the volume of pineal gland calcification

**DOI:** 10.1055/s-0046-1820103

**Published:** 2026-06-25

**Authors:** Carla A. Scorza, Fulvio A. Scorza, Josef Finsterer

**Affiliations:** 1 Universidade Federal de São Paulo, Escola Paulista de Medicina, São Paulo SP, Brazil.; 2 Neurology and Neurophysiology Center, Department of Neurology, Vienna, Austria.

Dear Editor,

We read with interest the article by Del Brutto et al. 1 on the correlation between the volume of pineal gland calcification determined using the SynthSeg 2 (open source) method and the counting of voxels > 50 HU in the study area and the Pittsburgh Sleep Quality Index (PSQI) in a cohort of 1,009 people from a community in Ecuador (Three Villages Study). Linear regression showed no positive correlation between the PSQI and the volume of pineal gland calcification. 1 The study is interesting, but some uncertainties still need clarification.

First, sleep quality depends not only on brain morphology but also on numerous internal and external influencing factors. 3 These include endogenous and exogenous factors such as genetic predisposition, personality type, stress management skills, comorbidities (such as restless legs syndrome, pain, and shortness of breath), sleep habits (such as bedtime, sleep aids [reading, television, music, and airing out the bedroom], removal and switching off of all devices that generate electromagnetic radiation), acute and chronic stress (such as type of work, noise, light, vibrations, drafts, insects, pets, earthquakes, type of bed, children, partners, electrosmog, nighttime light pollution, cell phone towers, relationships with neighbors, socioeconomic status, and local, regional, national, and geopolitical conditions), diet, time of last meal or fluid intake, concurrent medication use, consumption of alcohol, adrenergic stimulants or illicit drugs, and dietary supplements. 3

Secondly, sleep quality was assessed using the PSQI, a self-reported questionnaire based on seven components: subjective sleep quality, sleep latency, sleep duration, usual sleep efficiency, sleep disturbances, use of sleep aids, and daytime sleepiness. 4 However, the PSQI has some limitations in assessing sleep quality. 5 It is based on subjective assessments, correlates weakly with objective sleep measurements, yields inconsistent results across different populations, has limited validity for individuals with comprehension or writing difficulties, low test-retest reliability, and lacks guidelines on test-retest intervals. 5 Subjective assessments of sleep quality and sleep duration are imprecise and correlate weakly with objective measurements of sleep parameters.

The third point is that the patients did not undergo polysomnography. 1 To objectify the PSQI results, it would have been essential to confirm the sleep disturbances identified by the PSQI using polysomnography. Subjective assessments of sleep quality and duration can be misleading and lead to unreliable results. Therefore, PSQI data should be confirmed by objective measurement. Sleep quality and sleep patterns can be most reliably analyzed using polysomnography. 6

The fourth point is that pineal gland calcification is associated not only with sleep deprivation but also with age, environmental factors, diet, alcohol consumption, reduced sun exposure, Alzheimer's disease, ischemic stroke, and nicotine abuse. The volume of pineal gland calcification can depend on age, the extent of calcium deposition, the presence of comorbidities (such as attention deficit hyperactivity disorder, ADHD), the presence of pineal gland cysts, chronic use of beta-blockers, and lifestyle factors (such as smoking and level of schooling).

The fifth point concerns the question in the PSQI about whether a patient regularly takes sleep medication. However, it does not specify which medications the patient takes. 1 Knowledge of the specific medication is crucial, as efficacy and tolerability can depend heavily on the type of sleep medication and its dosage. How many patients regularly used illicit drugs such as cocaine, Bethel, or marijuana?

Sixth, the algorithm used to calculate the volume of pineal gland calcification was not specified. 1 Since the study analyzed the volume of pineal gland calcification, it is essential to know the method used to calculate it.

In sum, before concluding that volume of pineal gland calcification is not correlated with the PSQI, all factors influencing pineal gland size and the PSQI must be included in the multivariate regression analysis.
